# Large Carnivores Persisting in a Human‐Dominated Landscape: Suitable Habitat and Connectivity for Asiatic Black Bears in China

**DOI:** 10.1002/ece3.72181

**Published:** 2025-09-30

**Authors:** Jiale Cheng, Weicheng Zheng, Xiaohua Guo, Yu Wang, Yu Zhou, Shanshan Zhao, Xiao Song, Aichun Xu

**Affiliations:** ^1^ College of Life Sciences China Jiliang University Hangzhou China; ^2^ Suichang County Ecdogical Forestry Development Center Lishui China; ^3^ Administration Center of Zhejiang Jiulongshan National Nature Reserve Lishui China

**Keywords:** connectivity analysis, conservation, fragmentation, large carnivores, species distribution modeling

## Abstract

This study examined the connectivity between the current suitable habitat and the core habitat for Asiatic black bears ( *Ursus thibetanus* ) in human‐dominated landscapes and aimed to provide a basis for future conservation strategies for black bear populations in China. We collected occurrence locations (*N* = 130) of Asiatic black bears from 2014 to 2023, with data sources spanning 12 provincial administrative regions and covering an area of about 3,010,000 km^2^. We predicted the distribution of suitable habitats for Asiatic black bears via the MaxEnt species distribution model using a combination of multiple environmental variables such as topography, vegetation, climate, and anthropogenic disturbances. We performed habitat corridor planning by using the least‐cost path model and circuit theory. The results suggested that (1) Asiatic black bears have a marked preference for mountainous environments with middle and high altitude (> 1000 m), high amounts of precipitation (> 200 mm), and dense vegetation, and they generally avoid areas of anthropogenic disturbance. (2) The suitable habitats for Asiatic black bears showed a highly fragmented pattern, mainly concentrated along the borders of Zhejiang, Anhui, Fujian, and Jiangxi (the Zhe–Gan Region), the borders of Guangxi, Guangdong, and Hunan (the Hu–Guang Region), and the borders of Shaanxi, Chongqing, Hubei, Guizhou, and Sichuan (the Chuan–Shaan Region). Of the predicted 372,483 km^2^ of suitable habitat, only 23.65% is currently covered by protected and conserved areas. (3) A comprehensive landscape connectivity analysis identified 79 core habitat patches encompassing a total area of 33,257 km^2^. Notably, only 29.29% of these patches are currently under protection. Furthermore, we delineated 79 potential least‐cost paths, each with an average length of 43.66 km, and identified pinch points along these pathways that could impede connectivity. To ensure the long‐term survival of Asiatic black bear populations, we recommend enhancing the protection and restoration efforts for the three core habitats and their associated potential connectivity pathways.

## Graphical abstract

This study reveals that suitable habitats for Asiatic black bears in China are highly fragmented, with poor connectivity between core habitats, making conservation efforts urgently needed. Using the MAXENT model, the study predicted the distribution of suitable habitats and planned potential low‐cost migration corridors, providing a crucial basis for future conservation strategies for black bear populations.

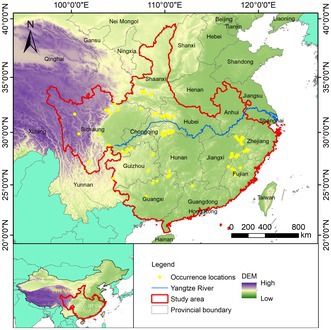

## 1 Introduction

Suitable habitats ensure the availability of core survival resources such as food, shelter, and breeding opportunities for wildlife, and thus are crucial to a species' persistence (Tellería 2016; Kosterman et al. 2018; Betts et al. 2019). Nevertheless, the rapid expansion of human activities has led to the loss, degradation, and fragmentation of natural habitats, making it one of the main threats to global biodiversity (Ceballos et al. 2015; Bogoni et al. 2022). This situation is especially severe for large carnivores and omnivorous species with similar space requirements, as they often require sizeable and connected habitats to sustain their populations (Wolf and Ripple 2017; Wang et al. 2024). Therefore, identifying and protecting suitable habitats for these species is significant for the conservation of biodiversity and the maintenance of ecosystem functions. Large carnivores and ecologically comparable omnivores face unprecedented challenges for survival due to their demand for large habitats and sensitivity to human disturbances (Su et al. 2021). Many of the world's large carnivore and wide‐ranging omnivore populations have experienced significant declines and are at risk of extinction (Hirt et al. 2021; Bodasing 2022). For example, as the only large carnivore in north China, the leopard ( *Panthera pardus* ) has undergone substantial reductions in its population range and numbers due to human activities (Wang et al. 2024). Correspondingly, the habitats of the Asiatic black bear ( *Ursus thibetanus* ), while omnivorous, show similar vulnerability patterns to strictly carnivorous species (Morovati et al. 2020). These situations not only reflect the environmental sensitivity of large carnivores but also emphasize the profound impact of human activities on natural ecosystems.

Despite these challenges, some large carnivores can survive in human‐dominated environments, primarily due to their behavioral plasticity and omnivorous characteristics that allow them to adapt to changing environmental conditions (Merkle et al. 2013; Chapron et al. 2014). For example, cases of coexistence between humans and large carnivores such as the wolf ( *Canis lupus* ) in Europe and North America and the African lion ( *Panthera leo* ) have shown that, through scientific management and thoughtful planning, humans and wildlife can coexist harmoniously (Schuette et al. 2013; Dorresteijn et al. 2014). These examples of successful coexistence provide valuable insights for protecting large carnivores in human‐dominated landscapes.

The Asiatic black bear, which is one of the key large carnivores in Asia, is capable of long‐distance dispersal (Yamazaki et al. 2008), has a significant impact on regional biodiversity and ecological balance. Nevertheless, owing to habitat loss and fragmentation, illegal hunting, and intensifying human activities, Asiatic black bear populations are greatly endangered (classified as Vulnerable on the IUCN Red List and a Class II protected species in China) (Escobar et al. 2015; Zahoor et al. 2021; Ahmad et al. 2022). The conservation and restoration of Asiatic black bear habitats face significant challenges, particularly in densely populated human areas, and there are occasional incidents of human–bear conflict (Ji et al. 2022; Hubbard et al. 2022). In recent years, although there has been a gradual increase in research on Asiatic black bears, landscape‐scale studies on key issues such as the distribution of suitable habitats, the connectivity of core habitats, and bears' responses to human activities remain inadequate (Jackson and Robertson 2011; Ali et al. 2023). Moreover, there is an urgent need for research on global issues such as the impact of climate change on Asiatic black bear habitat selection and the long‐term effects of land‐use changes on their population dynamics (Zahoor et al. 2021). In China, there are five subspecies of the Asiatic black bear (*U*. *t. thibetanus*, *U. t. laniger*, *U. t. mupinensis*, *U. t. formosanus*, and *U. t. ussuricus*), and the Sichuan subspecies (*U. t. mupinensis*) is the most widely distributed black bear in China. *U. t. mupinensis* is found in various provinces and regions, including Zhouzhi, Foping, and Taibai in Shaanxi Province, as well as Sichuan, Anhui, Zhejiang, Hubei, Hunan, Jiangxi, Leishan, and Fanjingshan in Guizhou Province, Guangxi, Guangdong, and Fujian (Gao 1987). This subspecies also faces the threat of habitat loss. Hence, in light of the widespread distribution of the Asiatic black bear and the severe threats it faces, it is imperative to conduct in‐depth research and implement effective conservation measures.

Based on Asiatic black bear distribution data obtained from infrared camera monitoring and news reports in the past decade, this study, which used the MaxEnt (maximum entropy) model (Phillips et al. 2006) to assess the suitable habitat for the Sichuan subspecies of the Asiatic black bear in China, identified crucial environmental factors influencing its distribution. Furthermore, the study used the least‐cost path (LCP) model and circuit theory to investigate potential ecological corridors and identify key areas limiting corridor connectivity. The findings provide data for assessing the status of Asiatic black bear populations and offer a scientific basis for conservation and restoration efforts.

## 2 Materials and Methods

### 2.1 Study Area

**FIGURE 1:**
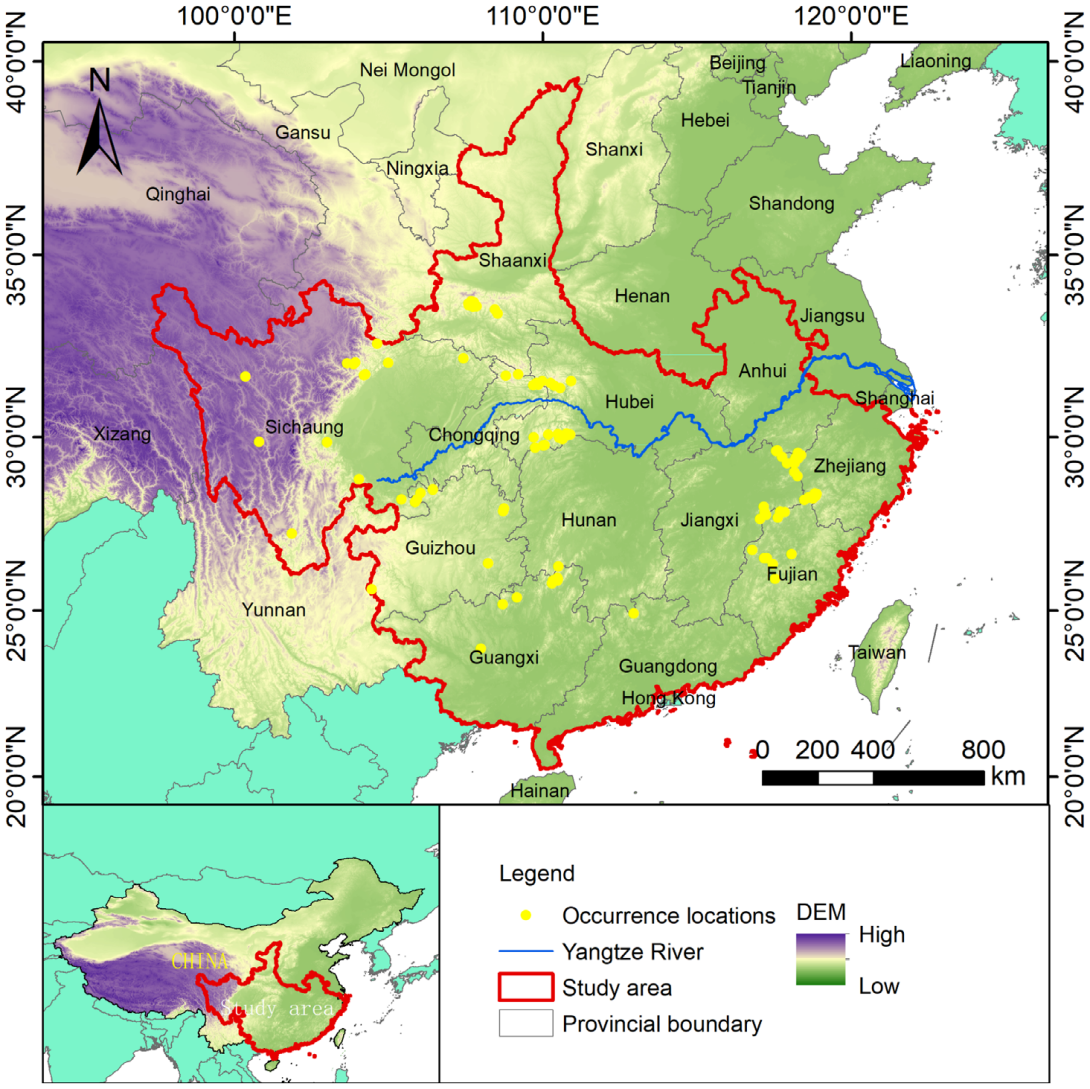
Study area and occurrence sites of the Asiatic black bear (*N* = 130) (DEM: Digital Elevation Model, data were obtained from the Geospatial Data Cloud (http://www.gscloud.cn)).

The study was carried out in 12 provinces in China (34°11′–43°49′ N, 103°11′–123°54′ E), covering an area of approximately 3,010,000 km^2^ (Figure 1), with 12 provincial‐level administrative units including Shaanxi Province, Sichuan Province, and Zhejiang Province. The region, located in the subtropical humid monsoon climate zone, is characterized by hot and rainy summers, relatively mild but dry winters, and an annual average temperature ranging between 15°C and 20°C. There is abundant annual precipitation, generally 800–1600 mm (China Meteorological Data Sharing Service System, http://cdc.cma.gov.cn/). The study area is characterized by diverse terrain, including high mountains, meandering rivers, and wide basins, with altitudes ranging from tens to thousands of meters above sea level (Chen et al. 2015). The dominant vegetation types in this region include subtropical evergreen broad‐leaved forests, coniferous forests, mixed forests, and bamboo forests (Wang et al. 2014, 2019). As a top predator, the Asiatic black bear plays an essential role in the ecosystem of this region. Not only is it a key species for maintaining the ecological balance of the forest but also an important indicator of forest health (Yamamoto, Oka, et al. 2012; Ali et al. 2017; Bashir et al. 2018).

### 2.2 Asiatic Black Bear Occurrence Data

Occurrence records of Asiatic black bears during the period from 2014 to 2023 were obtained from two sources—infrared camera trap surveys and public media reports. Using camera trapping survey data reported in the literature (Tian et al. 2020; Zheng et al. 2021; Dai et al. 2023) and other peer‐reviewed publications (Penteriani and Melletti 2020), we obtained occurrence locations of Asiatic black bears with precise coordinates. Furthermore, we performed searches on Baidu (China's largest search engine: www.baidu.com) and WeChat (China's most popular mobile messaging application) for reports of Asiatic black bear sightings or encounters from officially recognized news media. The criteria for including such reports included (1) the specific locations of Asiatic black bear sightings or encounters, and (2) photographs or videos of the sighted Asiatic black bears to ensure their reliability. The coordinates of the sighting locations were obtained using Omap (version 10.0.5).

### 2.3 Habitat Variables

According to previous studies, Asiatic black bears tend to select sunny and moderately sheltered ridges or slopes as their habitats. They use broad‐leaved forests and mixed coniferous and broad‐leaved forests with a high tree density, significant canopy cover, and steep slopes (Qi et al. 2011). To ensure a comprehensive assessment, given that habitat selection of Asiatic black bears has not been thoroughly studied in this region, we used 29 environmental variables as predictor variables for the Asiatic black bear habitat suitability model (Table 1). These included topographic variables (elevation, slope, and aspect), hydrological variables from the Geospatial Data Cloud (http://www.gscloud.cn), land use type variables from the Resource and Environmental Science and Data Platform (http://www.resdc.cn); climatic variables (rainfall, temperature, and sunshine) from the World Climate Database (http://www.worldclim.org), and human disturbance variables (settlements, roads, and cultivated land) derived from the 1:1 million geographic data for the year 2015 provided by the National Geomatics Center of China (http://www.ngcc.cn). Using ArcGIS (version 10.8), the raster size of the 29 environmental variable layers was uniformly resampled to 500 m × 500 m; the coordinate system was uniformly projected to WGS_1984_World_Mercator, and the layer boundaries were unified.

**TABLE 1:** Environment variables and the relevant information.

Variable	Describe	Unit
Bio1	Mean annual temperature	°C
Bio2	Mean diurnal temperature range	°C
Bio3	Isothermal property	ratio
Bio4	Standard deviation of the seasonal variation of temperature	SD
Bio5	Max temperature of the warmest month	°C
Bio6	Minimum temperature of the coldest month	°C
Bio7	Temperature annual range	°C
Bio8	Mean temperature of the wettest quarter	°C
Bio9	Mean temperature of driest quarter	°C
Bio10	Mean temperature of the warmest quarter	°C
Bio11	Mean temperature of the coldest quarter	°C
Bio12	Annual precipitation	mm
Bio13	Precipitation of the wettest month	mm
Bio14	Precipitation of the driest month	mm
Bio15	Precipitation seasonality	Coefficient of variation
Bio16	Precipitation of the wettest quarter	mm
Bio17	Precipitation of the driest quarter	mm
Bio18	Precipitation of the warmest quarter	mm
Bio19	Precipitation of the coldest quarter	mm
altitude	Altitude	m
aspect	Aspect (The absolute value of the actual slope aspect minus 180 degrees)	°
slope	Slope	°
d_water	Distance to water (Lakes, reservoirs, double‐lined rivers and ditches, etc.)	m
d_river	Distance to a river (Single‐line rivers, ditches, river structure lines, etc.)	m
d_house	Distance to a house	m
d_road	Distance to a road	m
d_rail	Distance to a railroad	m
NDVI	NDVI	
landuse	Land use (Forest land, Grassland, Cultivated land, Water, Residential land, Unutilized land)	Categorical Variable

### 2.4 Habitat Suitability Modeling

We considered two key factors: (1) absence data were only available from camera traps in Zhejiang province but not other regions, and (2) MaxEnt allowed effective integration of both systematic camera‐trap surveys and opportunistic media‐reported observations, so for habitat suitability modeling, we applied a presence‐background species distribution modeling framework using MaxEnt 3.3.3 k (Phillips et al. 2006), which is a program that creates predictive suitability models by comparing the values of environmental variables in presence cells with the values of the same variables in background cells. We used a standard presence‐background approach in MaxEnt, with background points randomly sampled from the entire study region. Although camera‐trap surveys in Zhejiang confirmed local absences, these data were not incorporated into the model. Occurrence data were randomly split into 75% training and 25% testing sets, bootstrapping was repeated 20 times, and Random seed was enabled; response curves and Jackknife were selected to evaluate the effects of environmental factors on species distribution, and other parameters were set by default. The output results are continuous raster data with values between 0 and 1 (Habitat Suitability Index, HSI), and we calculated Kappa statistics between bear occurrence points and 50% randomly selected MaxEnt background points across threshold intervals (0.1 increments), selecting the threshold that maximized Kappa value as the critical cutoff (Viña et al. 2010). Highly correlated bioclimatic variables (Pearson's |*r*| > 0.8) were removed. It is suitable for models using a small number of presence‐only records (Baldwin 2009; Valavi et al. 2022).

We reduced spatial autocorrelation by applying spatial rarefaction to occurrence points, implemented via the ‘Spatially Rarify Occurrence Data’ tool in SDMtoolbox (a GIS toolkit for species distribution modeling). Based on the minimum home range area of 5.1 km^2^ for Asiatic black bears (Ma et al. 1998), we used this tool to eliminate redundant points of Asiatic black bears that were within a distance of less than 1260 m (*r* = √[5.1/*π*]≈1.26 km). To assess potential bias from media‐sourced observations, we conducted a sensitivity analysis by comparing MaxEnt models trained on: (a) camera‐trap data only (subset analysis), and (b) all occurrence data. Model consistency was evaluated using Schoener's D niche overlap metric (Warren et al. 2010). All predictions represent relative suitability within the 12‐province study area rather than the species' full geographic range.

### 2.5 Landscape Connectivity Analysis

Based on the identified suitable habitats for Asiatic black bears within the study area, resistance factors impacting the movement of Asiatic black bears, as well as the weights and resistance values of each factor, were determined according to the degree of suitability. A resistance surface for Asiatic black bear movement was created using the raster calculator tool in ArcGIS 10.8 (Almasieh et al. 2019). Eight environmental variables (namely elevation, slope, aspect, distance to residential areas, distance to water sources, and distance to roads) were combined with certain weights to form a resistance surface (Qi et al. 2011; Li et al. 2019). The resistance values and weights for each environmental variable were assigned based on expert opinion and a review of species‐specific literature, with higher values representing greater movement resistance for bears. See Annex I for weights and resistance values. The comprehensive resistance surface (R) was then calculated using a weighted linear combination in the Raster Calculator tool in ArcGIS 10.8. Based on the “source” and resistance surface, an LCP analysis was employed to determine the minimum cost distance from each pixel to the “source” and establish the minimum cost path (Li et al. 2010). Habitat corridor planning was then conducted based on the results of the LCP analysis. Based on the species distribution modeling results, we delineated core habitat patches for Asiatic black bears by applying two ecologically meaningful thresholds: (1) areas exceeding the optimal HSI threshold (determined by maximum Kappa), and (2) a minimum patch size of 40 km^2^, which exceeds the typical home range size of an individual bear (5.1–36.5 km^2^) and supports a potential breeding group (Ma et al. 1998).

We modeled habitat corridors using the CorridorDesigner software in ArcGIS 10.8 (Almasieh et al. 2016). CorridorDesigner can identify the least‐cost corridors between endpoints. We determined an endpoint within each core area that encompassed all potential population patches within that core. Based on reports from local informants (direct observations, tracks, or scat) and the presence of high‐quality habitat or mountain ranges between potential pairs, we ensured that there was at least one simulated corridor originating from each core leading to its nearest neighboring core and we generated LCPs only between adjacent core habitats. We used the Pinchpoint Mapper Tool in Linkage Mapper, interfacing with Circuitscape (v. 4.0.7). This approach, which is based on the circuit theory (McRae and Beier 2007; McRae et al. 2008), identifies narrow sections within a corridor, known as pinch points. This analytical approach identifies narrow yet crucial areas within ecological corridors (Dickson et al. 2013). This analysis was applied across the entire study landscape to model omnidirectional connectivity and cumulative current flow, not solely within the pre‐defined LCPs. We then overlaid these pinch points onto LCPs to prioritize locations where corridor bottlenecks align with landscape‐wide connectivity constraints. These areas offer very limited choices in animal migration paths but are crucial to their movement. As a result, any disruption to these pinch points will have a disproportionate impact on the connectivity of the corridor (Dutta et al. 2016). Considering the current lack of clear guidelines for systematically identifying pinch points (Pelletier et al. 2014), we identified pinch points by visually identifying the darker (i.e., more purple) areas on the map produced by Circuitscape.

## 3 Results

A total of 130 Asiatic black bear locations were obtained through camera trapping surveys (114 locations) and media reports (16 locations) (Figure 1). After spatial autocorrelation testing, 116 locations were ultimately retained for analysis, with 100 camera‐trap‐only records used for locations for subset analysis.

### 3.1 Habitat Suitability

The ROC curve for the predicted suitable habitat model of Asiatic black bears within the study area provided a training set AUC = 0.975 and test AUC = 0.950. As this value is greater than 0.9, it is considered an “excellent” result (Zahoor et al. 2021), implying that the predictions made by the MaxEnt model are reliable and have high accuracy.

**FIGURE 2:**
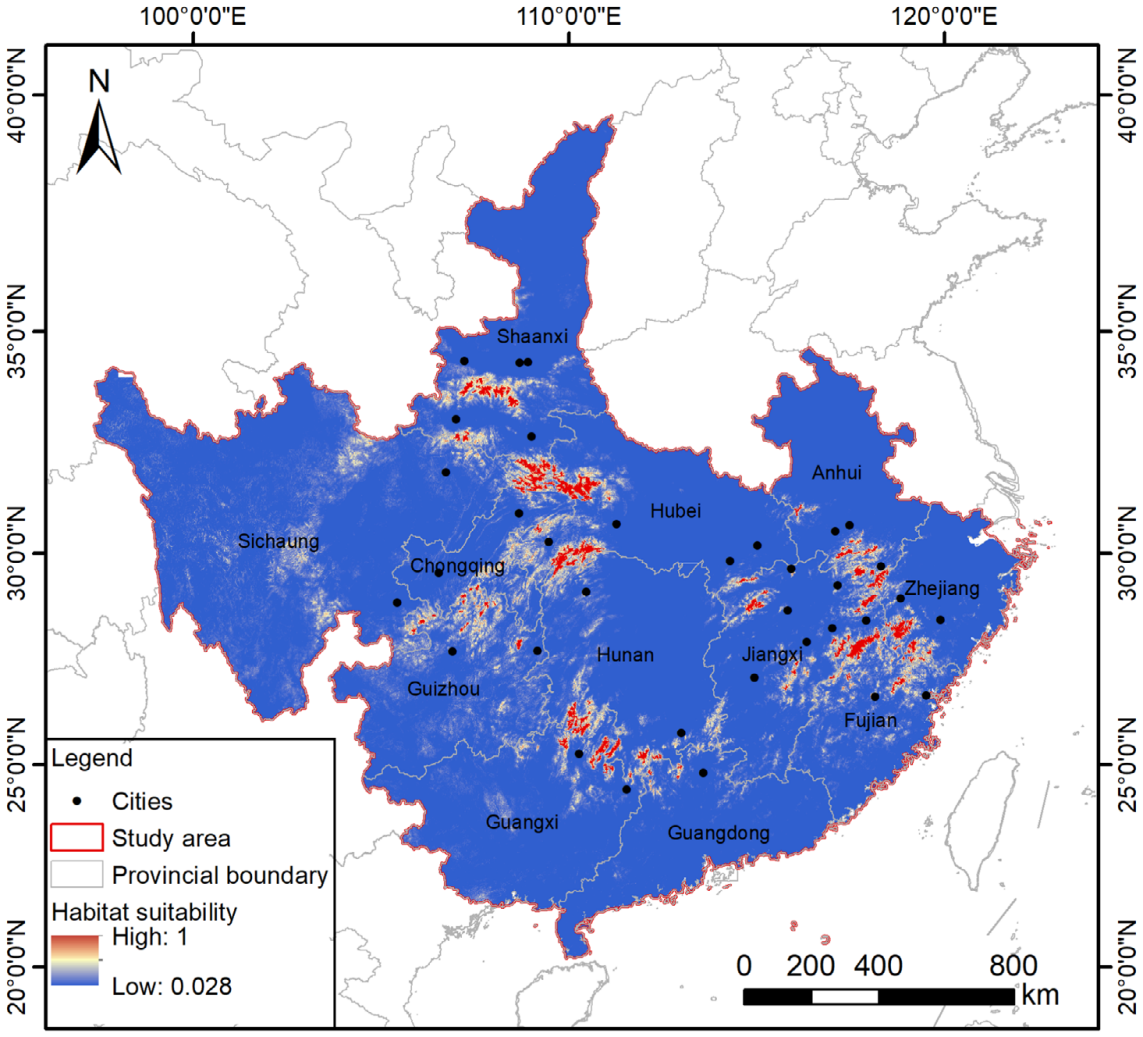
Habitat suitability predictions for the Asiatic black bear across its Chinese range (MaxEnt model; *n* = 29 variables; test AUC = 0.950). (Geographic scope: 12 provinces; suitability classification: 0–1 scale with HSI > 0.6 shown in red; base map: Elevation [DEM] with provincial boundaries; Cities: Urban permanent resident population exceeding 200,000. Data sourced from the Seventh National Population Census of China).

The prediction map revealed that the current suitable habitat for Asiatic black bears is highly fragmented, with large patches located in three key regions—along the borders of Zhejiang, Anhui, Fujian, and Jiangxi, the borders of Guangxi and Hunan, and the borders of Shaanxi, Chongqing, Hubei, Guizhou, and Sichuan (Figure 2). Of the 372,483 km^2^ of suitable habitat, only 43,035 km^2^ was classified as highly suitable habitat (HSI > 0.6). Kappa analysis showed that the maximum Kappa value was 0.544 at HSI = 0.6. Among the 142 patches of suitable habitat, 79 core habitat areas were identified, ranging in size from 41 to 3,922 km^2^, with an average area of 414.72 km^2^. The total area of these core habitats was 33,257 km^2^ (Table 2). Of these, 38 cores constituting 29.29% (9,741 km^2^) of the total core habitat area are existing protected areas (including 28 national (Table 2), seven provincial, one municipal, and two county‐level nature reserves), whereas the remaining 41 core habitat patches are not within protected areas. The presence of Asiatic black bears was confirmed in 17 of these core habitats, 14 of which overlap with protected areas.

**TABLE 2:** Area and proportion of suitable/unsuitable habitat for Asiatic black bears.

Category	Area (km^2^)	Proportion of study area	Covered by PC in km^2^ (%)
Unsuitable habitat	2,637,517	87.63%	144,594 (5.48)
Suitable habitat	372,483	12.37%	23,638 (6.35)
Core habitat	33,257	1.11%	9741 (29.29)

Abbreviation: PC, protected and conserved areas.

**FIGURE 3:**
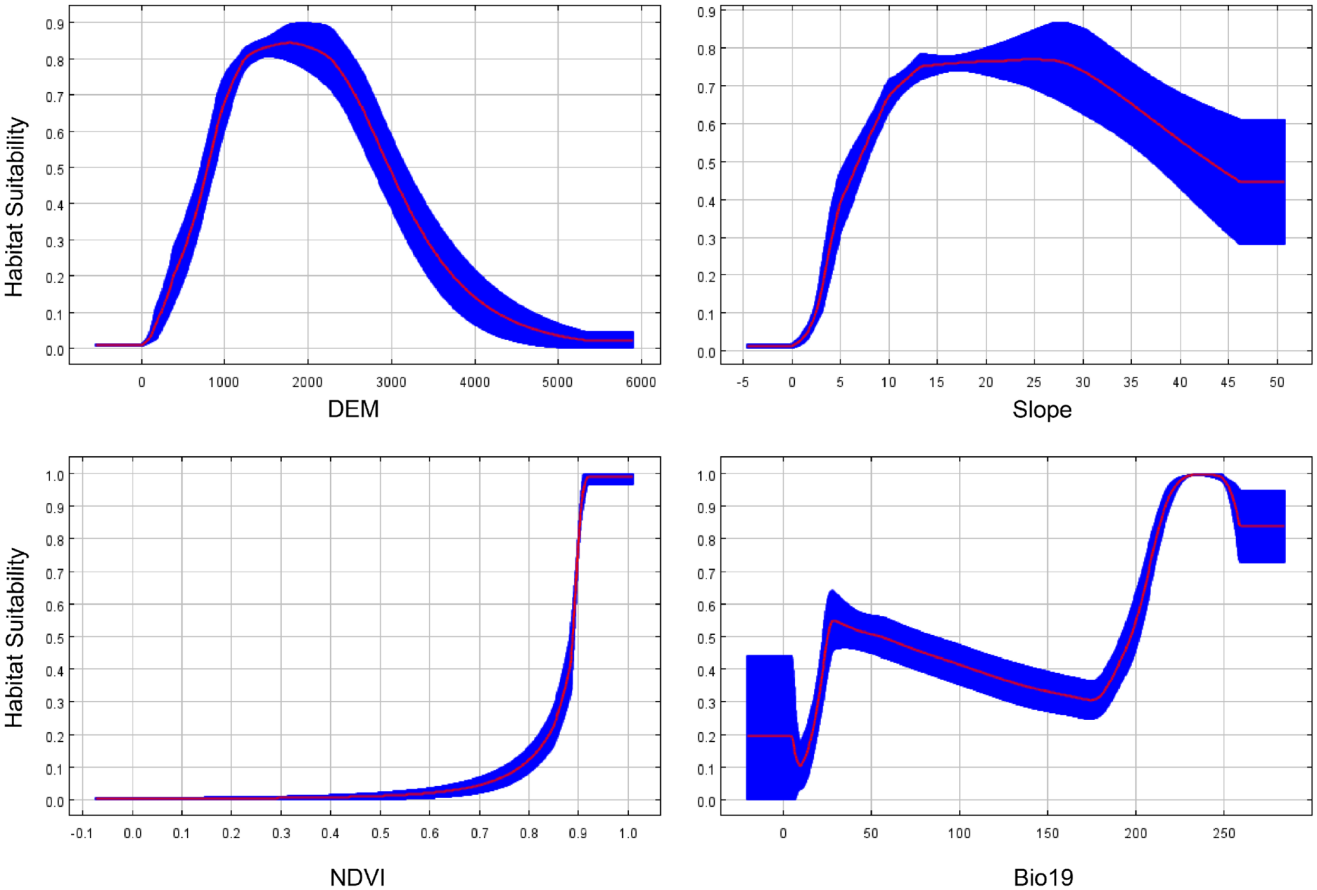
Response curves for the probability of the presence of Asiatic black bears (cloglog output from MaxEnt). Curves represent marginal effects of each variable while holding others at average values.

The average relative importance scores indicated that elevation, Bio19 (precipitation of the coldest quarter), slope, and normalized difference vegetation index (NDVI) were the primary factors influencing the suitability of Asiatic black bear habitats (Figure 3). The response curves showed that Asiatic black bears use middle and high elevations (> 1000 m) and high amounts of precipitation during the coldest quarter (> 200 mm), along with moderate slopes. The suitability of Asiatic black bear habitats showed a positive correlation with NDVI (Normalized Difference Vegetation Index), indicating the species used for areas with higher photosynthetic activity and canopy complexity.

Sensitivity analysis showed high consistency between models with and without media‐sourced observations (*D* = 0.804, *I* = 0.958), suggesting minimal bias from the inclusion of media data.

### 3.2 Landscape Connectivity

**FIGURE 4:**
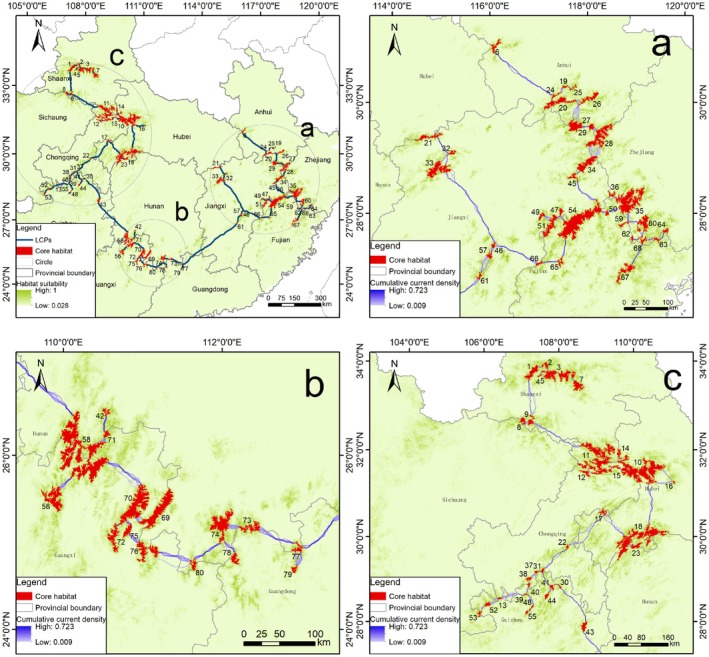
Landscape connectivity and pinch points for Asiatic black bears across the study area. The main panel shows core habitats, least‐cost paths, and the location of three key regions (outlined by circles a–c). The zoomed‐in panels below show details of the (a) Zhe‐Gan Region, (b) Hu‐Guang Region, and (c) Chuan‐Shaan Regions, with pinch points visible as dark purple areas within the corridors.

We identified 79 core habitat patches within the study area. The average Euclidean distance between all patches was 69.79 km (range: 3.16–378.10 km). We identified 79 LCPs among the 79 core habitats, with an average length of 43.66 km (range: 3.62–413.96 km). Two of the LCPs measured 249 km and 413 km. Dispersal events rarely exceed 200 km (Hellgren et al. 2005; Noyce and Garshelis 2011), and thus we divided the region into three areas: (a) the Zhe–Gan Region along the borders of Zhejiang, Anhui, Fujian, and Jiangxi; (b) the Hu–Guang region along the border of Guangxi and Hunan; and (c) the Chuan–Shaan region along the borders of Shaanxi, Chongqing, Hubei, Guizhou, and Sichuan (Figure 4). Numerous pinch points along potential corridors were underscored by our cumulative current density map (Figure 4), which may serve as critical corridors for the dispersal of Asiatic black bears.

## 4 Discussion

### 4.1 Key Factors Determining Habitat Suitability for Asiatic Black Bears

This study employed the MaxEnt model to analyze the impact of multiple environmental variables on the habitat suitability of Asiatic black bears. The results suggested that Asiatic black bears use mountainous environments with middle and high altitudes (> 1000 m), high precipitation (> 200 mm), and dense vegetation. Furthermore, the bears exhibit a certain degree of avoidance behavior toward human disturbance; however, the influence of human modification indices and roads was relatively minor compared to that of natural habitat characteristics. With their rich biodiversity, high‐altitude regions provide Asiatic black bears with a diverse range of food resources such as berries, nuts, and insects, fully meeting their dietary needs while also offering concealment and conditions for survival (Escobar et al. 2015; Zahoor et al. 2021). Moreover, the dense vegetation cover provides Asiatic black bears with concealment sites. It effectively protects Asiatic black bears from predation and human disturbance. Bashir et al. (2018) reported a strong positive correlation between the distribution of Asiatic black bears and the NDVI, highlighting the significance of vegetation cover in habitat selection for Asiatic black bears. The distribution of Asiatic black bears also displayed relative inconsistency with the slope of the study area, and this finding was supported by several previous studies (Zahoor et al. 2021; Goursi et al. 2021). Asiatic black bears in Japan tended to use steeper slopes (Takahata et al. 2013). This slope use may be based on the local production of grass, fruits, and seeds, which may be abundant on gentle slopes in the study area. The distribution and habitat utilization of Asiatic black bears show a degree of uncertainty, and their relationship with slope selection in the region is complex, necessitating future research on the trade‐offs between foraging efficiency, predation risk, and thermal cover on different slopes.

Habitat fragmentation results in the dispersion and isolation of species' habitats, thereby increasing the risk of extinction and potentially reducing gene flow between populations. These factors will affect the species' genetic diversity (Mondanaro et al. 2021). This study demonstrated a high degree of fragmentation of suitable habitats for Asiatic black bears across 12 provinces in China. This fragmentation has resulted in a scattered and discontinuous pattern of core habitats and thus poses a threat to the survival and reproduction of the black bear population. Among the 79 identified core habitats, only 29.29% (9741 km^2^) were effectively covered by existing protected areas, signifying that the majority of key habitats remain unprotected or inadequately protected. These suitable habitats that are not yet included in the protection system should also be given high priority and properly managed to ensure their preservation. Furthermore, habitat fragmentation may exacerbate conflicts between Asiatic black bears and humans (Oeser et al. 2023). Consequently, coexistence between humans and Asiatic black bears should be promoted using measures such as establishing ecological corridors, restoring habitats, and strengthening protection management.

Our sensitivity analysis demonstrated remarkably high consistency between models incorporating and excluding media‐sourced observations (D = 0.804, I = 0.958). This strong niche overlap suggests that the inclusion of these data points does not substantially alter our understanding of broad‐scale habitat suitability patterns. These findings align with growing evidence that carefully vetted citizen science data can provide valuable supporting information for species distribution modeling, particularly for wide‐ranging species where systematic surveys are logistically challenging (Rezvani et al. 2024; Wang et al. 2024).

### 4.2 Habitat Connectivity and Conservation Implications

The present study employed landscape connectivity analysis to identify 79 core habitat patches and their potential connectivity pathways. The results showed that the distribution of Asiatic black bears in China is narrow and island‐like, with suitable habitats being surrounded by densely populated areas including megacities like Chongqing (population > 15 million), regional hubs such as Baoji and Hanzhong, and ecologically sensitive edge cities (i.e., urban areas adjacent to protected zones where expansion threatens habitat connectivity), including Guilin, Shangrao, Quzhou, Nanping, and Huangshan (National Bureau of Statistics of China: http://www.stats.gov.cn). Considering the large home range of Asiatic black bears, averaging 5.1–36.5 km^2^ (Hou and Hu 1997; Ma et al. 1998), this narrow, island‐like distribution may be insufficient to meet the home range requirements necessary for population maintenance. Additionally, the island‐like pattern of habitat fragmentation may lead to edge effects, isolation effects, and crowding effects, further aggravating the extinction risk of Asiatic black bears (Almasieh et al. 2019). The majority of the identified patches with Asiatic black bear records are monitored within protected areas. For example, Patch 35 covering 1663 km^2^ (the Jiulongshan National Nature Reserve in Zhejiang, where at least six individuals have been identified (unpublished)), and Patch 3 spanning 746 km^2^ (the Foping National Nature Reserve) (Li et al. 2019; Ma et al. 2020) exceeded the home range sizes of individual Asiatic black bears reported in previous studies (Hwang et al. 2010; Yamamoto, Tamatani, et al. 2012; Immell et al. 2014). For patches where the presence of Asiatic black bears has not been confirmed, we recommend conducting infrared camera surveys to determine the current population status. Although there is currently a lack of direct evidence of Asiatic black bears in these areas, they could still serve as potential targets for reintroduction projects (Andersen et al. 2022).

Our connectivity analysis has revealed potential movement paths and seasonal stopovers during dispersals for Asiatic black bears between different habitats, a factor that is crucial for protecting their migration patterns. These corridors may facilitate not only individual dispersal but also population expansion by connecting isolated patches of high‐quality habitat (Betts et al. 2019). Nevertheless, we still know little about the actual use of these paths, especially those with pinch points. There were two ecological corridors stretching 249 km and 413 km between the three main distribution areas. However, considering records showing that three American black bears successfully migrated straight‐line distances of 282 km, 324 km, and 507 km (Rogers 1987; Stratman et al. 2001; Liley and Walker 2015), and considering the ecological and behavioral similarities between Asiatic black bears and American black bears (Fujiwara et al. 2013), we think it at least possible for bears to disperse, albeit rarely. Thus, these two ecological corridors may represent potential dispersal routes, but their actual use requires further verification. Highly dispersive/migratory animals can easily traverse areas that other animals typically avoid within their activity ranges (Keeley et al. 2016), signifying that managers may have considerable flexibility in determining the locations of ecological corridors. Among the corridors analyzed in this study, 25 were less than 10 km long and thus would incur low cost of protection. Nevertheless, these shorter corridors, especially those that can be crossed by animals in one or two days, should not be regarded as the sole appropriate conservation sites (Almasieh et al. 2016). Hence, the selection of corridors should consider whether they can meet the migration needs of highly mobile animals. The reason for this is that habitats of very low quality, such as mines, urban areas, or barren regions, may be unable to support animal dispersal. Owing to the insufficient research on the migration of Asiatic black bears, we suggest combining remote sensing studies with the tracking of a sample of Asiatic black bears via satellite collars (Rezvani et al. 2024). This approach can significantly enhance our understanding of functional connectivity in habitats and the prediction of corridors.

## 5 Conclusion

The present study used the MaxEnt model to systematically analyze the distribution and connectivity of suitable habitats for Asiatic black bears in China. Understanding the habitat use of Asiatic black bears in their choice of habitat. Further analysis showed the presence of potential low‐cost migration routes between core habitats. Offering a scientific basis for formulating conservation strategies for Asiatic black bears, this study highlights the importance of enhancing the protection of core habitats and migration routes.

## Author Contributions

**Jiale Cheng:** conceptualization (equal), data curation (equal), formal analysis (equal), investigation (equal), methodology (equal), project administration (equal), resources (equal), software (equal), supervision (equal), validation (equal), visualization (equal), writing – original draft (equal), writing – review and editing (equal). **Weicheng Zheng:** investigation (equal), supervision (equal), writing – review and editing (equal). **Xiaohua Guo:** methodology (equal), resources (equal), supervision (equal), validation (equal), writing – review and editing (equal). **Yu Wang:** investigation (equal), project administration (equal), resources (equal), supervision (equal). **Yu Zhou:** investigation (equal), supervision (equal), validation (equal). **Shanshan Zhao:** conceptualization (equal), data curation (equal), formal analysis (equal), funding acquisition (equal), investigation (equal), methodology (equal), project administration (equal), resources (equal), software (equal), supervision (equal), validation (equal), visualization (equal), writing – review and editing (equal). **Xiao Song:** conceptualization (equal), data curation (equal), formal analysis (equal), funding acquisition (equal), investigation (equal), methodology (equal), project administration (equal), resources (equal), software (equal), supervision (equal), validation (equal), visualization (equal), writing – review and editing (equal). **Aichun Xu:** conceptualization (equal), data curation (equal), formal analysis (equal), funding acquisition (equal), investigation (equal), methodology (equal), project administration (equal), resources (equal), software (equal), supervision (equal), validation (equal), visualization (equal), writing – review and editing (equal).

## Conflicts of Interest

The authors declare no conflicts of interest.

## Data Availability

Location, bioclimatic, and environmental variables for Asiatic black bears used in this paper are available via Dryad (https://doi.org/10.5061/dryad.2v6wwq002).

## References

[ece372181-bib-0001] Ahmad, F. , M. A. Nawaz , M. Salim , et al. 2022. “Patterns of Spatial Distribution, Diel Activity and Human‐Bear Conflict of *Ursus thibetanus* in the Hindu Kush Mountains, Pakistan.” Global Ecology and Conservation 37: e02145.

[ece372181-bib-0002] Ali, A. , Z. Zhou , M. Waseem , et al. 2017. “An Assessment of Food Habits and Altitudinal Distribution of the Asiatic Black Bear (*Ursus thibetanus*) in the Western Himalayas, Pakistan.” Journal of Natural History 51, no. 11–12: 689–701.

[ece372181-bib-0003] Ali, U. , B. Ahmad , R. A. Minhas , et al. 2023. “Habitat Suitability Modeling of Asiatic Black Bear (*Ursus thibetanus*) in Azad Jammu and Kashmir, Pakistan.” Pakistan Journal of Zoology 55, no. 3: 1273–1284.

[ece372181-bib-0004] Almasieh, K. , M. Kaboli , and P. Beier . 2016. “Identifying Habitat Cores and Corridors for the Iranian Black Bear in Iran.” Ursus (International Association for Bear Research and Management) 27, no. 1: 18–30.

[ece372181-bib-0005] Almasieh, K. , H. Rouhi , and S. Kaboodvandpour . 2019. “Habitat Suitability and Connectivity for the Brown Bear (*Ursus arctos*) Along the Iran‐Iraq Border.” European Journal of Wildlife Research 65, no. 4: 57–69.

[ece372181-bib-0006] Andersen, D. , Y. Yi , A. Borzée , et al. 2022. “Use of a Spatially Explicit Individual‐Based Model to Predict Population Trajectories and Habitat Connectivity for a Reintroduced Ursid.” Oryx : The Journal of the Fauna Preservation Society 56, no. 2: 298–307.

[ece372181-bib-0007] Baldwin, R. A. 2009. “Use of Maximum Entropy Modeling in Wildlife Research.” Entropy 11, no. 4: 854–866.

[ece372181-bib-0008] Bashir, T. , T. Bhattacharya , K. Poudyal , Q. Qureshi , and S. Sathyakumar . 2018. “Understanding Patterns of Distribution and Space‐Use by *Ursus thibetanus* in Khangchendzonga, India: Initiative Towards Conservation.” Mammalian Biology 92: 11–20.

[ece372181-bib-0009] Betts, M. G. , C. Wolf , M. Pfeifer , et al. 2019. “Extinction Filters Mediate the Global Effects of Habitat Fragmentation on Animals.” Science 366, no. 6470: 1236–1239. 31806811 10.1126/science.aax9387

[ece372181-bib-0010] Bodasing, T. 2022. “The Decline of Large Carnivores in Africa and Opportunities for Change.” Biological Conservation 274: 109724.

[ece372181-bib-0011] Bogoni, J. A. , K. M. P. M. B. Ferraz , and C. A. Peres . 2022. “Continental‐Scale Local Extinctions in Mammal Assemblages Are Synergistically Induced by Habitat Loss and Hunting Pressure.” Biological Conservation 272: 109635.

[ece372181-bib-0012] Ceballos, G. , P. R. Ehrlich , A. D. Barnosky , A. García , R. M. Pringle , and T. M. Palmer . 2015. “Accelerated Modern Human–Induced Species Losses: Entering the Sixth Mass Extinction.” Science Advances 1, no. 5: e1400253. 26601195 10.1126/sciadv.1400253 PMC4640606

[ece372181-bib-0013] Chapron, G. , P. Kaczensky , J. D. C. Linnell , et al. 2014. “Recovery of Large Carnivores in Europe's Modern Human‐Dominated Landscapes.” Science 346, no. 6216: 1517–1519. 25525247 10.1126/science.1257553

[ece372181-bib-0014] Chen, Y. Y. , W. Chen , W. Wu , C. M. Sun , T. Liu , and B. Z. Yang . 2015. “The Spatiotemporal Variation of Net Primary Productivity (NPP) of Vegetation in Southern China and Its Response to Climatic Factors.” Journal of Yangzhou University (Agricultural and Life Science Edition) 36, no. 3: 104–110.

[ece372181-bib-0015] Dai, Y. , H. Huang , Y. Qing , J. Li , and D. Li . 2023. “Ecological Response of an Umbrella Species to Changing Climate and Land Use: Habitat Conservation for Asiatic Black Bear in the Sichuan‐Chongqing Region, Southwestern China.” Ecology and Evolution 13, no. 6: e10222. 37384242 10.1002/ece3.10222 PMC10293704

[ece372181-bib-0016] Dickson, B. G. , G. W. Roemer , B. H. McRae , and J. M. Rundall . 2013. “Models of Regional Habitat Quality and Connectivity for Pumas ( *Puma concolor* ) in the Southwestern United States.” PLoS One 8, no. 12: e81898. 24367495 10.1371/journal.pone.0081898 PMC3867332

[ece372181-bib-0017] Dorresteijn, I. , J. Hanspach , A. Kecskés , et al. 2014. “Human‐Carnivore Coexistence in a Traditional Rural Landscape.” Landscape Ecology 29, no. 7: 1145–1155.

[ece372181-bib-0018] Dutta, T. , S. Sharma , B. H. McRae , P. S. Roy , and R. DeFries . 2016. “Connecting the Dots: Mapping Habitat Connectivity for Tigers in Central India.” Regional Environmental Change 16, no. 1: 53–67.

[ece372181-bib-0019] Escobar, L. E. , M. N. Awan , and H. Qiao . 2015. “Anthropogenic Disturbance and Habitat Loss for the Red‐Listed Asiatic Black Bear ( *Ursus thibetanus* ): Using Ecological Niche Modeling and Nighttime Light Satellite Imagery.” Biological Conservation 191: 400–407.

[ece372181-bib-0020] Fujiwara, S. , S. Koike , K. Yamazaki , C. Kozakai , and K. Kaji . 2013. “Direct Observation of Bear Myrmecophagy: Relationship Between Bears' Feeding Habits and Ant Phenology.” Mammalian Biology 78, no. 1: 34–40.

[ece372181-bib-0021] Gao, Y. T. 1987. Fauna of China: Mammalia, Volume 8, Carnivora. Science Press.

[ece372181-bib-0022] Goursi, U. H. , M. Anwar , L. Bosso , M. A. Nawaz , and M. Kabir . 2021. “Spatial Distribution of the Threatened Asiatic Black Bear in Northern Pakistan.” Ursus (International Association for Bear Research and Management) 2021: 1–5.

[ece372181-bib-0023] Hellgren, E. C. , D. P. Onorato , and J. R. Skiles . 2005. “Dynamics of a Black Bear Population Within a Desert Metapopulation.” Biological Conservation 122: 131–140.

[ece372181-bib-0024] Hirt, M. R. , A. D. Barnes , A. Gentile , et al. 2021. “Environmental and Anthropogenic Constraints on Animal Space Use Drive Extinction Risk Worldwide.” Ecology Letters 24, no. 12: 2576–2585. 34476879 10.1111/ele.13872

[ece372181-bib-0025] Hou, W. R. , and J. C. Hu . 1997. “The Status of Bear Resources and Their Conservation in China.” Journal of Sichuan Normal University (Natural Science) 4, no. 4: 17–21.

[ece372181-bib-0026] Hubbard, T. , M. V. Cove , and D. J. R. Lafferty . 2022. “Human Recreation Impacts Seasonal Activity and Occupancy of American Black Bears (*Ursus americanus*) Across the Anthropogenic‐Wildland Interface.” Scientific Reports 12, no. 1: 1–11. 35842446 10.1038/s41598-022-15665-x PMC9287820

[ece372181-bib-0027] Hwang, M.‐H. , D. L. Garshelis , Y.‐H. Wu , and Y. Wang . 2010. “Home Ranges of Asiatic Black Bears in the Central Mountains of Taiwan: Gauging Whether a Reserve Is Big Enough.” Ursus International Association for Bear Research and Management 21, no. 1: 81–96.

[ece372181-bib-0028] Immell, D. , D. H. Jackson , and M. C. Boulay . 2014. “Home‐Range Size and Subadult Dispersal of Black Bears in the Cascade Range of Western Oregon.” Western North American Naturalist 74, no. 3: 343–348.

[ece372181-bib-0029] Jackson, C. R. , and M. P. Robertson . 2011. “Predicting the Potential Distribution of an Endangered Cryptic Subterranean Mammal From Few Occurrence Records.” Journal for Nature Conservation 19, no. 2: 87–94.

[ece372181-bib-0030] Ji, Y. R. , L. S. Zhang , X. Y. Huang , et al. 2022. “Status of Human − Asiatic Black Bear Conflicts in Surrounding Communities of Baoshan Area in Yunnan Gaoligongshan National Nature Reserve.” Journal of Mammalogy 42, no. 4: 387–397.

[ece372181-bib-0031] Keeley, A. T. H. , P. Beier , and J. W. Gagnon . 2016. “Estimating Landscape Resistance From Habitat Suitability: Effects of Data Source and Nonlinearities.” Landscape Ecology 31, no. 9: 2151–2162.

[ece372181-bib-0032] Kosterman, M. K. , J. R. Squires , J. D. Holbrook , D. H. Pletscher , and M. Hebblewhite . 2018. “Forest Structure Provides the Income for Reproductive Success in a Southern Population of Canada Lynx.” Ecological Applications 28, no. 4: 1032–1043. 29457298 10.1002/eap.1707

[ece372181-bib-0033] Li, H. , D. Li , T. Li , Q. Qiao , J. Yang , and H. Zhang . 2010. “Application of Least‐Cost Path Model to Identify a Giant Panda Dispersal Corridor Network After the Wenchuan Earthquake—Case Study of Wolong Nature Reserve in China.” Ecological Modelling 221, no. 6: 944–952.

[ece372181-bib-0034] Li, R. , Y. M. Jiao , Y. Liu , Z. L. Liu , and X. Gao . 2019. “Evaluation of Suitable Habitat and Corridor Design for Green Peafowl Based on the MaxEnt Model.” Chinese Journal of Ecology 38, no. 3: 919–926.

[ece372181-bib-0035] Liley, S. G. , and R. N. Walker . 2015. “Extreme Movement by an American Black Bear in New Mexico and Colorado.” Ursus (International Association for Bear Research and Management) 26, no. 1: 1–6.

[ece372181-bib-0036] Ma, Y. Q. , L. Xu , and J. C. Hu . 1998. “Population Estimation of Bear Resources and Conservation Measures in China.” Life Science Research Journal 3: 52–59.

[ece372181-bib-0037] Ma, Y. S. , Q. Q. Ma , N. J. He , et al. 2020. “Surveying Mammal and Bird Diversity in Foping National Nature Reserve Using Infrared Camera Technology.” Biodiversity Science 28, no. 2: 226–230.

[ece372181-bib-0038] McRae, B. H. , and P. Beier . 2007. “Circuit Theory Predicts Gene Flow in Plant and Animal Populations.” Proceedings of the National Academy of Sciences 104, no. 50: 19885–19890. 10.1073/pnas.0706568104 PMC2148392 18056641

[ece372181-bib-0039] McRae, B. H. , B. G. Dickson , T. H. Keitt , and V. B. Shah . 2008. “Using Circuit Theory to Model Connectivity in Ecology, Evolution, and Conservation.” Ecology 89, no. 10: 2712–2724. 18959309 10.1890/07-1861.1

[ece372181-bib-0040] Merkle, J. A. , H. S. Robinson , P. R. Krausman , and P. Alaback . 2013. “Food Availability and Foraging Near Human Developments by Black Bears.” Journal of Mammalogy 94, no. 2: 378–385.

[ece372181-bib-0041] Mondanaro, A. , M. Di Febbraro , M. Melchionna , et al. 2021. “The Role of Habitat Fragmentation in Pleistocene Megafauna Extinction in Eurasia.” Ecography 44, no. 11: 1619–1630.

[ece372181-bib-0042] Morovati, M. , P. Karami , and F. Bahadori Amjas . 2020. “Accessing Habitat Suitability and Connectivity for the Westernmost Population of Asian Black Bear ( *Ursus thibetanus gedrosianus* , Blanford, 1877) Based on Climate Changes Scenarios in Iran.” PLoS One 15, no. 11: e0242432. 33206701 10.1371/journal.pone.0242432 PMC7673494

[ece372181-bib-0043] Noyce, K. V. , and D. L. Garshelis . 2011. “Seasonal Migrations of Black Bears ( *Ursus americanus* ): Causes and Consequences.” Behavioral Ecology and Sociobiology 65, no. 4: 823–835.

[ece372181-bib-0044] Oeser, J. , M. Heurich , S. Kramer‐Schadt , et al. 2023. “Prerequisites for Coexistence: Human Pressure and Refuge Habitat Availability Shape Continental‐Scale Habitat Use Patterns of a Large Carnivore.” Landscape Ecology 38, no. 7: 1713–1728.

[ece372181-bib-0045] Pelletier, D. , M. Clark , M. G. Anderson , B. Rayfield , M. A. Wulder , and J. A. Cardille . 2014. “Applying Circuit Theory for Corridor Expansion and Management at Regional Scales: Tiling, Pinch Points, and Omnidirectional Connectivity.” PLoS One 9, no. 1: e84135. 24497918 10.1371/journal.pone.0084135 PMC3907452

[ece372181-bib-0046] Penteriani, V. , and M. Melletti , eds. 2020. Bears of the World: Ecology, Conservation and Management. Cambridge University Press.

[ece372181-bib-0047] Phillips, S. J. , R. P. Anderson , and R. E. Schapire . 2006. “Maximum Entropy Modeling of Species Geographic Distributions.” Ecological Modelling 190, no. 3–4: 231–259.

[ece372181-bib-0048] Qi, Z. X. , W. H. Xu , X. Y. Xiong , Z. Y. Ouyang , H. Zheng , and D. X. Gan . 2011. “Assessment of Potential Habitat for *Ursus thibetanus* in the Qinling Moun‐Tains.” Biodiversity Science 19, no. 3: 343–352.

[ece372181-bib-0049] Rezvani, A. , M.‐R. Hemami , J. R. Goheen , et al. 2024. “Rethinking Connectivity Modeling for High‐Mobility Ungulates: Insights From a Globally Endangered Equid.” Landscape Ecology 39, no. 3: 73–88.

[ece372181-bib-0050] Rogers, L. L. 1987. “Effects of Food Supply and Kinship on Social Behavior, Movements, and Population Growth of Black Bears in Northeastern Minnesota.” Wildlife Monographs 97: 3–72.

[ece372181-bib-0051] Schuette, P. , S. Creel , and D. Christianson . 2013. “Coexistence of African Lions, Livestock, and People in a Landscape With Variable Human Land Use and Seasonal Movements.” Biological Conservation 157: 148–154.

[ece372181-bib-0052] Stratman, M. R. , C. D. Alden , M. R. Pelton , and M. E. Sunquist . 2001. “Long Distance Movement of a Florida Black Bear in the Southeastern Coastal Plain.” Ursus 12, no. 1: 55–58.

[ece372181-bib-0053] Su, H. , M. Bista , and M. Li . 2021. “Mapping Habitat Suitability for Asiatic Black Bear and Red Panda in Makalu Barun National Park of Nepal From Maxent and GARP Models.” Scientific Reports 11, no. 1: 14135. 34238986 10.1038/s41598-021-93540-x PMC8266906

[ece372181-bib-0054] Takahata, C. , S. Nishino , K. Kido , and S. Izumiyama . 2013. “An Evaluation of Habitat Selection of Asiatic Black Bears in a Season of Prevalent Conflicts.” Ursus (International Association for Bear Research and Management) 24, no. 1: 16–26.

[ece372181-bib-0055] Tellería, J. L. 2016. “Wildlife Habitat Requirements: Concepts and Research Approaches.” In Current Trends in Wildlife Research, edited by R. Mateo , B. Arroyo , and J. T. Garcia , 79–95. Springer International Publishing.

[ece372181-bib-0056] Tian, S. R. , H. W. Li , B. W. Jiang , Y. M. Chen , Y. Li , and D. Q. Li . 2020. “Spatial and Temporal Patterns of Mammalian Diversity in the Huubuanshan National Nature Reserve, Hunan, China.” Journal of Mammalogy 40, no. 1: 87–95.

[ece372181-bib-0057] Valavi, R. , G. Guillera‐Arroita , J. J. Lahoz‐Monfort , and J. Elith . 2022. “Predictive Performance of Presence‐Only Species Distribution Models: A Benchmark Study With Reproducible Code.” Ecological Monographs 92, no. 1: e01486.

[ece372181-bib-0058] Viña, A. , M.‐N. Tuanmu , W. Xu , et al. 2010. “Range‐Wide Analysis of Wildlife Habitat: Implications for Conservation.” Biological Conservation 143, no. 9: 1960–1969.

[ece372181-bib-0059] Wang, J. , K. L. Wang , M. Y. Zhang , and Y. F. Duan . 2014. “Analysis of Spatial and Temporal Variations of NDVI and Its Driving Factors in the Southern Hilly Mountain Belt.” Resources Science 36, no. 8: 1712–1723.

[ece372181-bib-0060] Wang, W. M. , C. M. Li , J. W. Shu , and W. Chen . 2019. “Changes in Vegetation Cover in Southern China.” Chinese Science: Earth Science 49, no. 8: 1308–1320.

[ece372181-bib-0061] Wang, Y. , M. Liu , F. Xia , et al. 2024. “Big Cats Persisting in Human‐Dominated Landscape: Habitat Suitability and Connectivity of Leopards in Central North China.” Landscape Ecology 39, no. 5: 94–108.

[ece372181-bib-0062] Warren, D. L. , R. E. Glor , and M. Turelli . 2010. “ENMTools: A Toolbox for Comparative Studies of Environmental Niche Models.” Ecography 33, no. 3: 607–611.

[ece372181-bib-0063] Wolf, C. , and W. J. Ripple . 2017. “Range Contractions of the World's Large Carnivores.” Royal Society Open Science 4, no. 7: 170052. 28791136 10.1098/rsos.170052 PMC5541531

[ece372181-bib-0064] Yamamoto, T. , T. Oka , N. Ohnishi , H. Tanaka , N. Takatsuto , and Y. Okumura . 2012. “Genetic Characterization of Northernmost Isolated Population of Asian Black Bear ( *Ursus thibetanus* ) in Japan.” Mammal Study 37, no. 2: 85–91.

[ece372181-bib-0065] Yamamoto, T. , H. Tamatani , J. Tanaka , et al. 2012. “Annual and Seasonal Home Range Characteristics of Female Asiatic Black Bears in Karuizawa, Nagano Prefecture, Japan.” Ursus (International Association for Bear Research and Management) 23, no. 2: 218–225.

[ece372181-bib-0066] Yamazaki, K. , C. Kozakai , S. Kasai , Y. Goto , S. Koike , and K. Furubayashi . 2008. “A Preliminary Evaluation of Activity‐Sensing GPS Collars for Estimating Daily Activity Patterns of Japanese Black Bears.” Ursus 19, no. 2: 154–161.

[ece372181-bib-0067] Zahoor, B. , X. Liu , L. Kumar , Y. Dai , B. R. Tripathy , and M. Songer . 2021. “Projected Shifts in the Distribution Range of Asiatic Black Bear ( *Ursus thibetanus* ) in the Hindu Kush Himalaya due to Climate Change.” Ecological Informatics 63: 101312.

[ece372181-bib-0068] Zheng, W. C. , Z. Q. Chen , Z. H. Zheng , et al. 2021. “Monitoring of Black Bear Activities in Jiulongshan National Nature Reserve, Zhejiang Province, and Prediction of Its Potential Distribution Area in East China.” Chinese Journal of Zoology 56, no. 4: 509–521.

